# Management of Testicular Silicone Gel-Filled Prosthesis Rupture: Case Report of a Rare Event and a Review of the Literature

**DOI:** 10.1155/2016/2824802

**Published:** 2016-03-30

**Authors:** Quang-Bao Phan, Nicolas Koutlidis, Céline Duperron, Eric Mourey, Frédéric Michel, Luc Cormier

**Affiliations:** ^1^Department of Urology, University Hospital François Mitterrand, 14 rue Paul Gaffarel, 21000 Dijon, France; ^2^Department of Urology, William Morey Hospital, Chalon sur Saône, 4 rue Capitaine Drillien, 71321 Chalon-sur-Saône, France

## Abstract

*Introduction*. We report a case of spontaneous rupture of a single testicular prosthesis in a patient who had undergone bilateral orchiectomy and silicone gel-filled prosthesis insertion. The consequences of this rare event are discussed. There is no management algorithm.* Case Presentation*. A 55-year-old man presented to our outpatient department with altered consistency in his right testicular prosthesis and a painful right hemiscrotum with no systemic symptoms thirty-three years after the implantation of the prosthesis. We removed this implant without replacement, in accordance with the patient's wishes.* Conclusion*. The long time between the implantation and the spontaneous rupture is remarkable and was never before described. The removal of the prosthesis was straightforward and it would have been possible to implant a new prosthesis after taking into account the condition of the skin.

## 1. Introduction

Many circumstances, including testicular cancer and a necrotic testicle due to spermatic cord torsion, can lead to orchiectomy.

A testicular implant is commonly inserted in adults in the absence of a peroperative scrotal skin wound after radical orchiectomy. This simple procedure provides patients with a cosmetically normal scrotum and reduces the psychological impact of testicle loss [1, 2]. These prostheses are well accepted by patients [3, 4]. Since 1973, silicone gel-filled implants have been used to obtain a naturally feeling testis: the implants comprise an outer silicone elastomer which envelops a transparent silicone gel [5, 6].

Since the 1990s and the well-documented complications with silicone gel-filled breast implants and their theoretical health risks, such as connective tissue and autoimmune disorders or the possibility of tumor development [7], silicone elastomer or saline-filled prostheses have been now used [8, 9].

Early complications include hematoma, wound dehiscence, or infection. Late complications are ilioinguinal neuralgia, discomfort, or pain and complications of implants (mainly displacement to a higher position, extrusion, or rupture) [10]. Spontaneous implant rupture is a very rare event.

## 2. Case Presentation

**Figure 1:**
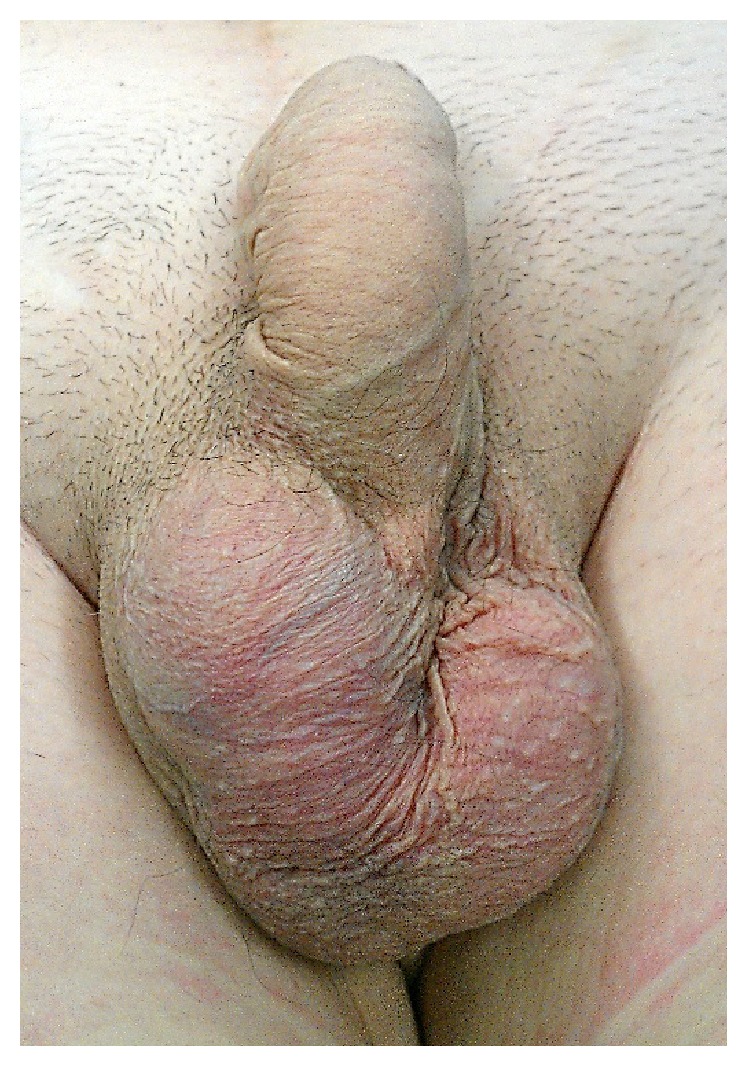
Scrotum.

A 55-year-old man presented to our outpatient department with a three-month history of increased right hemiscrotum volume. He reported discomfort when sitting. There was no history of trauma and there were no systemic symptoms. Examination revealed a swollen and painful right hemiscrotum with a loss of consistency of the right testicular prosthesis. The scrotum content appeared to be stuck to the skin, and the overlying skin was rough with asperities (Figure 1). No regional adenopathy was detected.

The past history was marked by right testicular teratoma at the age of 22 with a left undescended testicle. He was treated with right radical orchiectomy and adjuvant chemotherapy (three cycles of bleomycin, actinomycin, cisplatin, and vinblastine). The patient then underwent retroperitoneal lymph-node dissection for residual masses (no metastases were found at histology) followed by six months of adjuvant treatment (cyclophosphamide, methotrexate, and actinomycin). This treatment was stopped for intolerance.

During the follow-up, hypogonadism appeared and was treated with testosterone replacement therapy. Six months later, the patient underwent left radical orchiectomy with concomitant bilateral testicular prosthesis insertion via the inguinal approach because of an undescended hypoplastic, nonfunctional left testicle. No intraoperative puncture of the implants was mentioned. Follow-up since insertion had been unremarkable.

**Figure 2:**
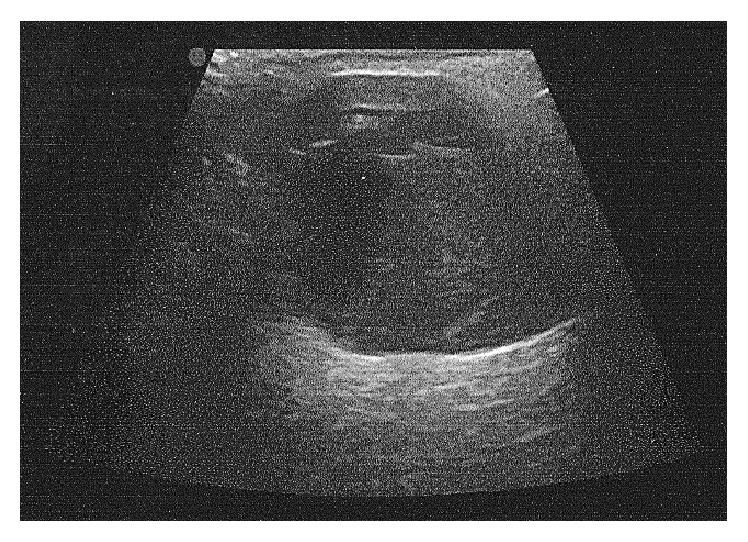
Scrotal ultrasonography with right ruptured implant and collapsed envelope.

At presentation, the biological investigations were normal. Scrotal ultrasonography (US) revealed a normal left testicular prosthesis. The right prosthesis seemed ruptured with septation echoes due to the device shell (Figure 2).

**Figure 3:**
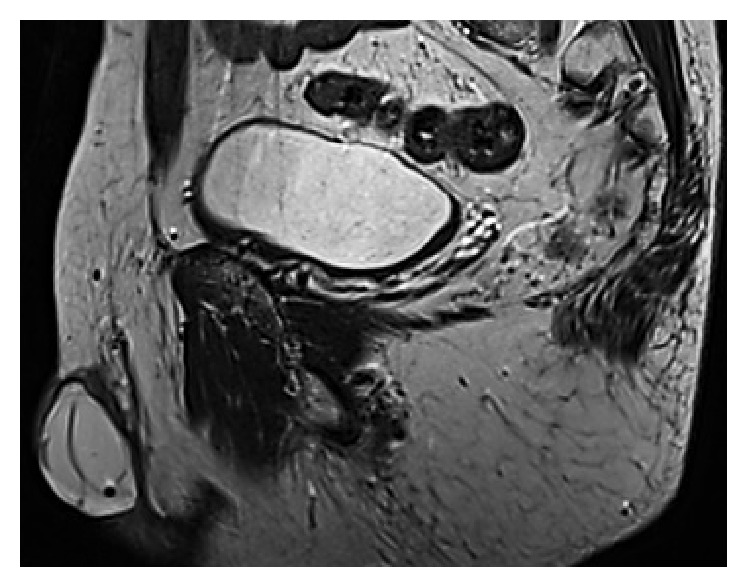
MRI showing prosthesis with linguini sign (free-floating loose thread).

Magnetic resonance imaging (MRI) showed no suspect scrotal tissues but confirmed the rupture of the right implant, which presented multiple intraprosthetic septations (Figure 3).

**Figure 4:**
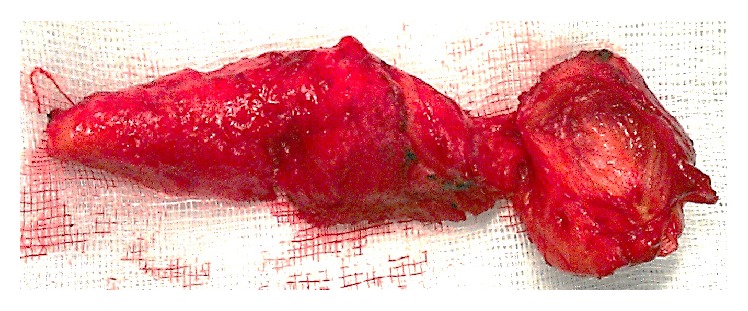
Siliconome and the implant capsule.

In view of the symptoms and in accordance with the patient's wishes, the right prosthesis was surgically removed without replacement. A scrotal approach was chosen. The skin incision released the silicone gel which had spread into the scrotum. After the removal of the ruptured prosthesis, the implant capsule and the surrounding fibrosis were also removed (Figure 4) and the altered scrotal skin was excised. Follow-up was uneventful.

Histology showed only inflammatory tissues: giant cell reaction to foreign material. Bacteriological analysis of the implant was negative.

## 3. Discussion

In spontaneous testicular implant rupture, the clinical signs are scrotal or perineal pain, erythematous rash, pruritus, altered consistency of the prosthesis, or palpation in the hemiscrotum of an irregular soft mass [11, 12]. The patient presented rough overlying skin with asperities and scrotal pain. To our knowledge, there is no case of spontaneous prosthesis rupture with palpable inguinal adenopathy to be reported.

Breast silicone gel-filled implant failure is much more frequent and has been more frequently described. The detection of rupture in testicular silicone gel-filled prostheses is based on that in breast implants and the same techniques and terms are used. Rupture can be confirmed by US and MRI. The term “stepladder sign” was described in breast implant rupture: it is the most reliable evidence of rupture on US [13]. It corresponds to the septa echoes within the implant due to the collapsed envelope of the ruptured implant. The term “linguini sign” (or free-floating loose thread) in MRI was first used by Gorczyca et al. to describe the multiple curvilinear hypointense lines within the high signal-intensity silicone filling of the ruptured implant [14]. In our case, the stepladder sign was incomplete. It is a useful but inconstant sign on US evaluations [13, 15]. This is why MRI, which is more reliable, was described as the imaging method of choice for suspected testicular implant rupture [11].

Although there were no locoregional signs, we removed the implant because the patient was symptomatic. The patient declined replacement prosthesis. Hage et al. reported an easy implant substitution procedure in the absence of symptoms of scrotal inflammation, by using the cavity provided by the fibrous capsule once it had been cleaned or removed [11]. However, a conservative approach has also been described in a patient with no locoregional disease to avoid a new scrotal exploration [16].

Spontaneous testicular prosthesis rupture is a rare event, whereas, for breast implants, it occurs in up to 77% of the cases [17, 18]. Only two cases of spontaneous testicular prosthesis rupture were found in the literature [12, 16]. According to Hage et al., it may be caused by chronic intermittent trauma. The incidence of rupture may depend on the location of the implant. The scrotum, unlike subpectoral sites, may protect the implant from pressure injury as it allows greater mobility and less friction [11, 17].

According to experience with breast implants, silicone gel can occasionally migrate to distant sites. In rare cases, it leads to neurovascular bundle damage, granuloma, and cyst formation, or the breakdown of overlying tissues. The capsule of fibrous tissue around the implant results from a foreign body reaction, which leads to the production of specific protein antibodies to the prosthesis components. Nevertheless, there is no scientific evidence of systemic effects [17].

Late clinical complications in ruptured testicular prosthesis are ilioinguinal neuralgia, discomfort or pain, scrotal contraction, and complications of implants (mainly displacement to a higher position, extrusion, or rupture with silicone leakage) [10–12]. No association has been found between late local or systemic complications and silicone testicular implants [19, 20]. Moreover, there are no reported cases in the literature of tumors arising from the use of silicone testicular implants [9]. Transcapsular migration of silicone particles has been observed even when the fibrous capsule was intact [21, 22]. There have been no reports of associations between leakage of silicone gel from testicular prostheses and adverse effects. However, we should probably remove all ruptured prostheses to avoid or to treat local symptoms especially as the procedure is simple. However, silicone gel-filled prostheses do not need to be removed systematically for prophylactic purposes as their rupture is a very rare complication. Patients with such implants should have long-term follow-up or at least regular self-palpation [23].

## 4. Conclusion

Unilateral rupture was described in a patient with bilateral prosthetic testes, but our case differs as the spontaneous rupture occurred 33 years after implantation, which was subsequent to orchiectomy for neoplasia. We removed the implant and scrotal skin as the patient was symptomatic. There was no replacement. All ruptured prostheses should be removed; others should be followed-up.
